# Age influences the distribution of diffuse gliomas

**DOI:** 10.18632/aging.203414

**Published:** 2021-08-09

**Authors:** Alexandre Roux, Pascale Varlet, Johan Pallud

**Affiliations:** 1 Service de Neurochirurgie, GHU Paris – Hôpital Sainte-Anne, Paris, France; 2 Université de Paris, Sorbonne Paris Cité, Paris, France; 3 Inserm UMR 1266, IMA-BRAIN, Institut de Psychiatrie et Neurosciences de Paris, Paris, France; 4 Service de Neuropathologie, GHU Paris – Hôpital Sainte-Anne, Paris, France

**Keywords:** diffuse gliomas, alioblastomas, age, histo-molecular, epigenetic

Diffuse gliomas are the one of the most common malignant primary brain tumours in paediatric, adolescent and young adult (AYA), and adult patients [1]. Age is a main factor influencing the occurrence of cerebral gliomas and, particularly, the histo-molecular profile varies with age [2]. Interestingly, when we analyze the histo-molecular subtypes of diffuse gliomas in children <15 years, AYAs (i.e. between 15 and 25 years), adults between 25 and 55 years, and adults >55 years, we observe a variation of the distribution of the histo-molecular subtypes according to the age group analyzed (Figure 1). *H3K27*-mutant diffuse gliomas represent the most frequent subgroup in the paediatric population (33%), *H3G34*-mutant diffuse gliomas have a higher frequency in AYAs (13%), *IDH*-mutant diffuse gliomas have a higher frequency in adults between 25 and 55 years (60%), and *IDH*-wildtype diffuse gliomas represent the most frequent molecular subgroup in adults >55 years (58%) [1, 2].

**Figure 1:**
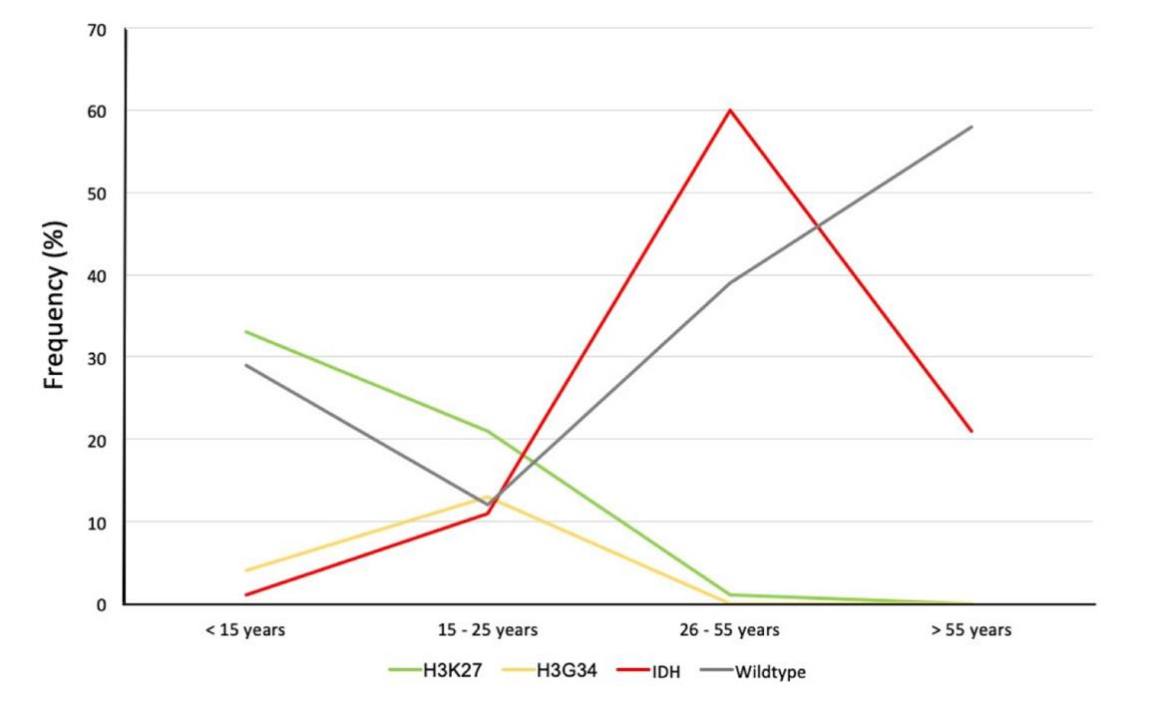
**Age distribution of histo-molecular subtypes of diffuse gliomas.** Green: Diffuse midline gliomas, H3K27-mutant; Yellow: Diffuse gliomas, H3G34-mutant; Red: Diffuse gliomas, IDH-mutant (with and without 1p/19q-codeletion); Grey: Diffuse gliomas, IDH-wildtype.

Age also influences the spatial distribution of *IDH*-wildtype glioblastomas. In patients ≥60 years, they more likely involve the cerebral hemispheres (frontal, temporal, parietal, and insular lobes more than occipital and limbic lobes) [3]. Contrarily, in younger patients, they more likely involve the midline (diencephalo-mesencephalon and thalamic regions) [3].

Age is a main prognostic factor for survival of diffuse gliomas, whatever the histo-molecular subtypes, together with the overall condition of the treatment, the extent of the surgical resection, and the adjuvant neuro-oncological treatments. Moreover, age influences the neurosurgical treatment, including the decision as to whether to perform a large surgical resection [4]. Indeed, although the extent of surgical resection is associated with a significant improvement in progression-free and overall survivals whatever the age of the patient, the rates of postoperative complications and the impact of surgery on functional independence possibly increases with age and is particularly a concern in the elderly population [4]. Similarly, the feasibility of adjuvant neuro-oncological treatments are influenced by age [5, 6]. For instance, the standard radiochemotherapy protocol for *IDH*-wildtype glioblastomas (i.e. 60Gy in 30 fractions with concurrent chemotherapy with Temozolomide followed by 6 cycles of adjuvant Temozolomide) is validated for patients <70 years. For older patients with functional independence (i.e. Karnofsky performance status >70%), a concentrated radiochemotherapy protocol (40Gy in 15 fractions) is proposed [6]. In the other cases, a chemotherapy alone or supportive care are proposed. In the same way, many radiotherapy and chemotherapy protocols for diffuse gliomas have been tried in paediatric patients [5], with very different patterns from those used in adults.

Recently, epigenetic ages [7] and aging-related genes [8] have provided promising results, including their use as prognostic factors for diffuse glioma patients. Liao et al. have demonstrated that epigenetic ages analyzes yield insights into coherent modifications of the epigenome related to different subtypes of gliomas, and have shown correlations with survival and recurrence [7]. Xiao et al. have analyzed the expression profiles, the prognostic value, and the potential mechanisms of action of aging-related genes in diffuse gliomas [8]. They designed risk scores and cluster models based on aging-related genes and glioma cases. This scoring system was correlated with malignant clinical features, poor prognosis, and genomic aberrations of aging-related oncogenes [8].

Altogether, all available data in literature highlight the impact of age on clinical presentation, location, histo-molecular subtype, neurosurgical and neuro-oncological treatments, epigenetic ages, and aging-related genes for diffuse gliomas. It appears essential to merge all these data to better define the role of age in the occurrence and evolution of diffuse gliomas. Age by itself should not be the sole criterion to guide the neuro-oncological treatment and such merge analyzes will allow to choose, on an individual basis, the best therapeutic options for a particular patient. It will contribute to improve patients’ survival and to reduce the under-treatment we observe in elderly patients.

## References

[1] Ostrom QT, et al. Neuro Oncol. 2020; 22:iv1–v96. 10.1093/neuonc/noaa200 33123732 PMC7596247

[2] Roux A, et al. Neuro Oncol. 2020; 22:1190–202. 10.1093/neuonc/noaa024 32025728 PMC7594566

[3] Roux A, et al. Radiology. 2019; 293:633–43. 10.1148/radiol.2019190491 31592732

[4] Zanello M, et al. J Neurooncol. 2017; 135:285–97. 10.1007/s11060-017-2573-y 28726173

[5] Hargrave D, et al.. Lancet Oncol. 2006; 7:241–48. 10.1016/S1470-2045(06)70615-5 16510333

[6] Perry JR, et al. N Engl J Med. 2017; 376:1027–37. 10.1056/NEJMoa1611977 28296618

[7] Liao P, et al. Neuro Oncol. 2018; 20:942–53. 10.1093/neuonc/noy003 29432558 PMC6007761

[8] Xiao G, et al. Aging (Albany NY). 2021; 13:13239–63. 10.18632/aging.203008 33946049 PMC8148480

